# Immunotherapeutics in Multiple Myeloma: How Can Translational Mouse Models Help?

**DOI:** 10.1155/2019/2186494

**Published:** 2019-04-10

**Authors:** Rachel E. Cooke, Rachel Koldej, David Ritchie

**Affiliations:** ^1^Australian Cancer Research Foundation (ACRF) Translational Laboratory, Royal Melbourne Hospital, Melbourne, Australia; ^2^Department of Medicine, University of Melbourne, Melbourne, Australia

## Abstract

Multiple myeloma (MM) is usually diagnosed in older adults at the time of immunosenescence, a collection of age-related changes in the immune system that contribute to increased susceptibility to infection and cancer. The MM tumor microenvironment and cumulative chemotherapies also add to defects in immunity over the course of disease. In this review we discuss how mouse models have furthered our understanding of the immune defects caused by MM and enabled immunotherapeutics to progress to clinical trials, but also question the validity of using immunodeficient models for these purposes. Immunocompetent models, in particular the 5T series and Vk^⁎^MYC models, are increasingly being utilized in preclinical studies and are adding to our knowledge of not only the adaptive immune system but also how the innate system might be enhanced in anti-MM activity. Finally we discuss the concept of immune profiling to target patients who might benefit the most from immunotherapeutics, and the use of humanized mice and 3D culture systems for personalized medicine.

## 1. Introduction

Multiple myeloma (MM) is a malignancy of plasma cells that reside within a supportive niche in the bone marrow (BM) [1, 2]. Monoclonal gammopathy of undetermined significance (MGUS) is a preceding, benign phase to MM, where a monoclonal paraprotein is detected in the peripheral blood but plasma cells account for less than 10% of BM haematological cells [3, 4]. Smoldering myeloma (SMM) is similarly asymptomatic, but plasma cells account for at least 10% of BM haematological cells. Patients are often diagnosed with MM when they develop end-organ features that include anaemia, bone fractures secondary to lytic lesions, hypercalcaemia, and/or renal disease [1, 2]. Acquired immune paresis complicates advanced disease due to residual hypogammaglobulinemia, B cell hypoplasia [5], the effects of cumulative chemotherapies [6–8], and an ageing T cell population [9, 10]. In end stage disease, plasma cells lose their dependence on the BM niche and can cause extramedullary disease with solid organ deposits and/or plasma cell leukaemia.

MM is a disease of older adults with a peak incidence in the 7^th^ decade of life [11]. The increasing use of proteasome inhibitors and immunomodulatory drugs (IMiDs) over the last decade has made an impact on overall survival in MM patients [12, 13] but has transformed MM to a chronic palliative illness. As our knowledge of immunosenescence and T cell exhaustion within the chronic inflammatory environment of MM advances, evaluating the effectiveness of immunotherapeutics within a tumor microenvironment in an aged host is paramount. This review aims to encompass how mouse models can contribute to our understanding of the MM immune microenvironment and of the clinical use of immunotherapeutics and other novel agents in human MM.

## 2. Mouse Models of Multiple Myeloma

The two main types of mouse models used (Table 1) include

1. immunodeficient xenograft models where mice lack immune subsets rendering them tolerant to the transplant of human MM cells (often referred to as “humanized”),
2. immunocompetent mice that are either transgenically manipulated to develop a MM-like tumor or transplanted with MM cells from a syngeneic mouse.

### 2.1. Immunodeficient Models

SCID (severe combined immunodeficiency) and NSG (NOD/SCID/IL2R*γ*^null^) mouse models inoculated with human MM cells are widely used for drug discovery; however, their obvious disadvantage is that neither immunosurveillance nor the tumor microenvironment that supports myeloma cell survival is fully intact. Regardless, xenograft models are frequently used to assess antimyeloma therapies including monoclonal antibodies and vaccines.

#### 2.1.1. Human MM Cell Lines

MM cell lines are derived from clones from humans with plasma cell leukaemia or other forms of extramedullary disease. These cells have evolved to survive outside of the bone marrow niche, have complex cytogenetic profiles, and are highly resistant to apoptosis. They have a rapid doubling time of 24-72 hours and are therefore very easy to culture. But for all these reasons, they do not accurately resemble most human myeloma cells, which are typically very difficult to culture outside of human BM. MM cell lines can be injected into mice intravenously via the tail vein, intratibially, or subcutaneously (SC). The former two methods are preferred as they more faithfully represent BM disease in MM, whereas SC injection imitates solitary plasmacytoma in an entirely separate immune compartment. Whilst cell lines are likely to be selective for highly effective antimyeloma therapies, their use in an immunodeficient setting does not recapitulate the typical immune myeloma microenvironment and may not allow an opportunity for immunotherapies to fully exert their effect.

### 2.2. Immunocompetent Models

The most commonly used immunocompetent mouse models of multiple myeloma in the literature are the 5T series and transgenic* myc*-overexpressing models (or syngeneic transplanted cell lines from these diseased mice).

The 5T series (including 5T2MM, 5T33MM, and 5TGM1) are cell lines derived from aged C57BL/KaLwRij mice that spontaneously developed plasma cell dyscrasia. On syngeneic transplant, recipients develop dissemination of the tumor, paraprotein, osteolytic bone lesions, and resultant hind limb paralysis [14, 15].

Knowledge of driver mutations in MM led to the development of the Vk*∗*MYC [16] and Myc/Bcl-X_L_ [17]* myc*-overexpressing mouse models of myeloma, and the E*μ*-XBP-1s model [18]. All spontaneously develop MM-like tumor dissemination and paraprotein over a longer latency, with greater heterogeneity of disease than single clonal transplant. However, the time to disease is a greatly limiting factor at the bench. Syngeneic transplant of cells retrieved from diseased Vk*∗*MYC mice has similar MM-like disease but with faster kinetics [16], and the myeloma cells are responsive to most conventional therapies used in humans [19].

Less commonly used now are the plasmacytoma-resembling murine MM cell lines (MOPC315, J558, HOPC) that have been transplanted subcutaneously in syngeneic mice. These were obtained from granulomas arising from the intraperitoneal injection of mineral oil in Balb/c mice. The cell line MOPC315.BM has been derived from MOPC315 cells that exhibit bone marrow tropism [20].

## 3. The Tumor Microenvironment and Immune Dysfunction in MM

It is well established that MM cells influence the BM microenvironment to sustain tumor survival. This is achieved by pathologies that include osteoclastogenesis, increased angiogenesis, and immune editing. The role of xenograft and 5T murine models of MM to assess the efficacy of therapeutic agents for bone disease was reviewed recently [21].

Immunosurveillance describes the processes by which the immune system recognizes and eliminates foreign pathogens and tumor cells. This theory has been refined over the last 15 years to the concept of “immune editing”, which is a dynamic process composed of three phases: (1) elimination, (2) equilibrium, and (3) escape [22]. In MM, the equilibrium phase is most noteworthy as it represents a therapeutic opportunity to utilize the immune system to slow or prevent disease progression. Immunosurveillance has been demonstrated in the Vk*∗*MYC mouse model, where immune control of MM was demonstrated via NK and CD8^+^ T cells through CD226 (DNAM-1) interaction with its ligand CD155 on malignant plasma cells [23].

The development of immunosenescence, through which age-related changes of immune system lead to functional defects, may also contribute to loss of immunosurveillance with subsequent progression of tumors. These age-related changes include a drastic loss of thymic function and a decrease in the number and repertoire of naïve T cells in the 7^th^ decade [24, 25], coinciding with the peak incidence of MM. Simultaneously, there exists a chronic inflammatory state termed inflammaging: a sustained, low-grade increase in proinflammatory factors such as IL-6, IL-1, TNF*α*, and CRP [26]. This adds to the increased susceptibility of older humans to opportunistic infections, cancer, and autoimmunity [27]. Some of these changes are seen, and possibly accelerated, in malignancy and/or chronic viral infections, as discussed below.

### 3.1. Adaptive Immune System

This section concentrates on T cell pathology in MM that has been most intensively studied in the immunotherapeutics field. B cells have predominantly been evaluated in mouse models with regard to the oncogenic mutations that promote development into plasma cell malignancy. B cell hypoplasia has been described in human MM [5] and in the Vk*∗*MYC mouse model [28], and further study of how this might affect anti-MM T cell function is warranted.

#### 3.1.1. T Cell Generation

Thymic involution leads to an age-associated decrease in the frequency of circulating naïve T cells in peripheral blood (PB) [10, 29], lymph nodes [30], and bone marrow (BM) [31]. Of additional relevance to the MM patient population, it has been shown that the human thymus is incapable of responding to a sudden decline in peripheral T cells (i.e., after high dose chemotherapy or radiation) with a substantial increase in T cell output [32]. Studies with bone marrow transplant patients have shown that the thymus of the majority of patients over 40 years was unable to rebuild a naïve T cell compartment [33].

Despite the reduction in thymic output, overall T cell numbers are not affected due to compensatory proliferation of T cells in the periphery [34]. However, CD4^+^ T cells do not proliferate to the same degree as CD8^+^ T cells [33, 35] leading to a reduction in CD4:8 in MM patients [28, 36], which could be partly explained by the higher expression of CD122 (the *β*-chain of the IL-2/IL-15 receptor) on CD8^+^ T cells [37] and increased availability of IL-15 in lymphopenic states [34]. Additionally, IL-7 dependent STAT-1 activation has been reported to limit homeostatic CD4^+^ T cell expansion [38], and naïve CD8^+^ T cells are particularly hyperresponsive to IL-15 because of lack of suppressor of cytokine signaling (SOCS)-1 [39]. The emergence of an oligoclonal T cell population with a limited TCR repertoire has been observed [40], as well as a senescence-associated secretory phenotype (SASP) that has low proliferative potential but retains the ability to produce cytokines and does not exhibit telomere shortening that is seen with ageing populations [40, 41].

The loss of naïve T cell populations is not paralleled in aged mice, where the thymus sustains the naïve T cell pool throughout their lifetime [42], and the CD4:8 ratio remains unaffected in diseased Vk*∗*MYC mice [28]. One study showed an impaired ability of aged mice to thymically recover T cells after irradiation [43], although in most cases this state is not replicated in MM mouse models because mice used in experimental models are invariably young adults. Of interest, it has been shown in the Vk*∗*MYC transplant model that CD8^+^CD44^+^ T memory cells were integral to MM control after BM transplant; however, mice transplanted with naïve (CD44^−^) T cells had improved survival, indicating the importance of naïve T cell priming [44].

#### 3.1.2. T Cell Differentiation

In addition to reduced thymic output of naïve T cells, chronic antigen exposure leads to alterations in the proportion of naïve: antigen-experienced T cells. This has been described in humans with ageing, persistent viral infections, and chronic malignancy. A major skewing towards a T cell population predominantly made up of effector memory T (T_EM_) and CD8^+^  T_EMRA_ cells has been demonstrated in human MM and replicated in Vk*∗*MYC mice with advanced disease in both the transplant and transgenic models [28]. This pattern has also been noted in another model of chronic B cell malignancy, the E*μ*TCL1 mouse model of chronic lymphocytic leukaemia [45].

#### 3.1.3. T Cell Exhaustion

T cell exhaustion refers to an altered T cell state that is manifested under conditions of chronic inflammation, such as chronic viral infection or cancer [46]. Exhausted T cells are not inert; but the loss of effector functions limits their ability to fully eradicate pathogens or tumor. CD8^+^ T cells expressing inhibitory markers correlated strongly with disease progression after BM transplant in the Vk*∗*MYC mouse model [47]. Knowledge of inhibitory T cell signaling pathways has been instrumental in developing immunotherapeutics such as PD-1 and CTLA-4 inhibitors that are currently in human trials (see Therapeutics).

#### 3.1.4. T Cell Polarization

Several groups have published evidence that there are increased numbers of Th17 cells in the PB and BM microenvironment of patients with MM compared to normal [48–50], and elevated levels of IL-17 [49, 50] and Th17-polarizing cytokines (IL-6, TGF*β*, IL-23, and IL-1*β*) [49] in the BM. This has been proposed to be harmful in MM by promoting lytic bone disease [49, 51] and MM cell growth [50]. Others have suggested that the Treg/Th17 balance is the important factor, and lower Treg numbers carry a favorable prognosis [52]. Studies in Treg levels and activity have also been contradictory [53, 54], which is further confusing as to how to define Tregs by flow cytometry [52, 55, 56]. This remains an area for further exploration as greater understanding of the epigenetic factors involved in T cell polarization and the potential for plasticity between the subsets is developed [57].

In the mouse models, there is a notable Th1 response as evidenced by increased IFN*γ* production (predominantly by CD8^+^ T cells) with advanced disease in Vk*∗*MYC mice [28, 58]. Transition from a Th1 to a Th2 response with increased IL-4 and IL-13 production has been described with advanced disease in the transgenic Vk*∗*MYC model [58], and Th2 cells provided no protection against disease in a 5TGM1 transplant model (and may even promote MM growth by promoting VEGF production) [59]. Th17 cells and IL-17 production was not significantly altered in Vk*∗*MYC mouse models [28], but it would be of interest to assess this in longstanding disease correlating with amount of bone disease and relative proportion of Tregs. Later work in the Vk*∗*MYC model has been suggestive of a pathological role for IL-17: IL-17A deficient donor grafts and inhibition of IL-17A with mAb improved MM control after BM transplant and, conversely, donor derived IL-17A promoted MM cell survival [44].

Increased Treg populations were described in the spleen, lymph nodes, bone marrow, and peripheral blood of 5T2 and 5T33 transplant mouse models, and these cells retained their suppressive function* ex vivo* [60]. In further analysis in the 5T2 model, it was evident that there are temporal differences in Treg accumulation, with changes being observed early in the spleen and peripheral blood but only at later stages of the disease in bone marrow.

### 3.2. Innate Immune System

Innate immune responses occur without prior exposure to antigen and memory T cell formation. Cells considered part of the innate immune response include granulocytes, antigen-presenting cells (APCs) such as dendritic cells (DCs), natural killer (NK) cells, and unconventional T cells such as invariant natural killer T (iNKT) cells and *γδ* T cells. The latter make up a more substantial and diverse proportion of the murine immune system than in humans [61]. All of these cells have been described to be adversely affected in human MM [62–67] and are selectively discussed in more detail in Therapeutics.

Type I interferons are cytokines produced after immune cell recognition of pathogen-specific molecules via pattern recognition receptors such as Toll-like receptors (DCs can be prolific producers). Release of type I interferons has numerous effects but is overall stimulatory to T cells by causing upregulation of MHC I and II on cells and hence increased peptide presentation. The consequences of drug-induced type I interferon production are discussed in DC Vaccines and Small Molecule Inhibitors.

Myeloid-derived suppressor cells (MDSCs) are immature myeloid cells that are increased in inflammatory states and play a pathological role in cancer by suppressing effector T cell function and promoting Treg expansion [68, 69]. They have been described as fundamental to MM-associated immunosuppression in the Vk*∗*MYC MM model and are driven by IL-18 that has emerged as a potential therapeutic target [70].

## 4. Matching Models with Human MM

### 4.1. Disease Stage

Chromosomal instability begins with MGUS, and cumulative chromosomal changes occur throughout the course of disease [71]. Secondary translocations, including dysregulation of c-myc, occur later in disease as the tumor becomes addicted to oncogenes [72] and escapes immunosurveillance. It would therefore seem logical that myc-overexpressing mouse models might better represent advanced disease, and those models that lack c-myc oncogene rearrangements (5TMM [73], XBP-1) might provide an opportunity to study the aetiopathogenesis of MM, in particular how MGUS transforms to MM. In all cases, it should be considered whether these models truly have an MGUS period, or whether they represent an initial state akin to smoldering MM with steady accumulation of tumor until mice exhibit symptomatic disease.

In our experience with the Vk*∗*MYC mouse model, it was important to interpret data in context of the amount of tumor burden and to take into account the differing disease dynamics of the transgenic and transplant models [28]. As an example, there have been contradictory reports in Vk*∗*MYC mice of either BM accrual of CD4^+^ and CD8^+^ T cells with increasing disease [58] or depletion of CD8^+^ T cells with advanced disease [23] that can be accounted for by substantially different disease burdens in these cases. We found that immune dysfunction in Vk*∗*MYC mice with advanced disease was in keeping with relapsed/refractory multiple myeloma (RRMM) in humans [28], which certainly remains an area of need for novel therapeutics. Only using models with aggressive disease, however, could lead to agents being overlooked that work in indolent disease when there is a more functional immune system. Hence, if the focus of research is in preventing disease progression in the MGUS or smoldering phase of disease, aged transgenic mice with disease arising* de novo* are likely to provide a better model.

### 4.2. Cell Compartments

A valid criticism of translational studies is of the comparisons made between different cell compartments in mouse models and human samples. For obvious reasons, spleen and BM samples are not readily available from humans, and serial blood samples are most accessible for studies of immune cells. Where comparisons have been made between PB and BM mononuclear cells in human MM, CD4^+^ subsets and associated cytokine profiles have been similar [28, 48–50], although PB contamination of BM samples does occur. There are differences in a few parameters however; for example, CD4:8 ratio is higher in PB than BM and there are fewer CD4^+^  T_CM_ and more T_EMRA_ in BM than PB (which correlates with lower CD27 and higher CD57 expression in BM CD4^+^ cells) [28].

Unlike humans (where extramedullary haematopoiesis is abnormal), the spleen is considered a haematopoietic organ in mice [74] and most frequently used for T cell analysis in studies because of ease of access and increased numbers of T cells retrievable. In many of the MM mouse models, hepatosplenic plasma cell infiltration and/or plasmacytomas occur and it is unclear whether this should be accepted as equivalent to BM infiltration or rather as true extramedullary disease.

## 5. Therapeutics

Much of the preclinical experimentation with immunotherapeutics has been performed in immunodeficient mice (Tables 2 and 3). Xenograft mouse models have proven useful in providing preclinical data for the use of novel immunotherapies in phase 1 human trials. Additionally, where drugs that looked favorable in the* in vitro* setting failed to yield sufficient clinical responses in phase 1 and 2 trials, returning to these models has helped provide evidence for combination therapies and phase 3 trials in humans. As already alluded to, xenograft models only provide proof of concept for the therapeutic efficacy of immunotherapeutics, and their effect in humans is often much more subdued than that in preclinical trials. Performing experiments in both immunodeficient and immunocompetent mice has been integral in elucidating the mechanism of action of novel agents (see Small Molecule Inhibitors).

### 5.1. Cellular Therapies

The oldest form of cellular therapy, stem cell transplantation, has been reviewed recently in MM [75]. However, chimeric antigen receptor (CAR)-T cells have really captured the scientific and public attention of late. Another approach to enhance anti-MM cytotoxic T cell activity is via dendritic cell (DC) vaccination, although DCs are significantly dysfunctional in MM patients [62, 63] that have repercussions for effective vaccination.

#### 5.1.1. CAR-T Cells

CAR-T cells are cytotoxic T cells engineered to express receptors specific for a target antigen. In adoptive immunotherapy, millions of these cells are cultured in the laboratory and administered to the patient intravenously. For a broader review of the history and evolution of CAR-T cells in MM, readers are directed to other review articles [76, 77].

CAR-T constructs have been created for use in MM against B cell maturation antigen (BCMA), CD19, and kappa light chains. Whilst a 100% cure rate was achieved in xenograft murine models with anti-BCMA constructs [78, 79], only very modest effects have been achieved in phase 1 trials in humans [80, 81]. Engineered NK cells specific to CD138 [82] and CS-1 [83] have also been effective* in vitro *and* in vivo* mouse models of human MM.

Because immunodeficient mouse models have been used in preclinical work, CAR-T cell-induced cytokine release syndrome (a not uncommon feature in humans) cannot be predicted for. This is an advantage in that significant morbidity and mortality might be avoided in the mice, but means that the human immune response is not being faithfully replicated: we are essentially measuring the ability of CAR-T cells to reach their target antigen in an* in vivo* system and perform cytolysis (as they would in an* in vitro* setting).

Treating MM patients with CAR-T cells remains a long way from widespread use clinically, mainly because of the cost but also the challenge of producing an effective and persistent T cell product from elderly and/or heavily pretreated patients. Studies of CD19 CAR-T cells in an NSG mouse model of lymphoma have shown that T_N_ and T_CM_ produce a superior CAR-T product to T_EM_ in terms of cytokine production (CD4^+^) and cytotoxicity (CD8^+^), and the potency of CD8^+^ CAR-T cells is enhanced by their production in the presence of CD4^+^ T cells [84]. It would therefore seem logical to collect and sort CD62L^+^ T cells (i.e., T_N_ and T_CM_) for CAR-T production from MM patients at first diagnosis, prior to T cell depleting therapies and subsequent further skewing of the T cell population, even if they are not used until relapse.

#### 5.1.2. DC Vaccines

DC vaccines are produced from autologous* ex vivo* DCs generated from PB monocytes or BM progenitor cells that are exposed to MM-specific antigens. These can be derived from MM lysates or dying MM cells, or DCs can be transfected with MM-derived RNA or fused directly with MM cells. The goal of DC priming is, via enhancement of tumor-specific antigen presentation, to stimulate tumor-specific cytotoxic T cell activity and overcome T cell tolerance.

In MOPC-315 plasmacytoma-bearing mice, DC vaccine in combination with IMiDs controlled plasmacytoma growth [85]. Unfortunately this does not bear out in humans, where DC vaccines frequently show antigen-specific immune responses but do not demonstrate tumor regression [86, 87]. Returning to mouse models may yet provide an insight into how to improve clinical outcomes by enhancing DC function through choice of progenitor cell, cytokine stimulation or priming antigen, and timing and route of administration and by rescuing defective DC function (reviewed from a broader oncological perspective recently [88–92]). As an example, 5T33MM mice inoculated with *α*-GalCer-loaded DCs moderately prolonged survival [93]. Therapies that promote maturation of DCs and enhance type I interferon may also prove useful: for example, the novel Toll-like receptor agonist C792 inhibited plasmacytoid DC-induced MM cell growth in a xenograft model and enhanced the effectiveness of antimyeloma therapies [94].

A logical combination therapy with DC vaccines is checkpoint blockade (see Section 5.2.3), because PD-L1/2 expressed on DCs can be associated with suppression of effector T cells and expansion of Tregs [63]. It has been suggested that DC vaccination might be better utilized at a shorter interval after, or concurrently with, chemoradiotherapy to optimize immunogenic cell death, as suppressive immune cells are at their lowest at this time. Furthermore, their use in the posttransplant setting could be influential in the reemerging lymphocyte population. This is discussed further in the next section.

### 5.2. Monoclonal Antibodies (MoAbs)

Monoclonal antibodies in the treatment of MM have been developed to target the plasma cell itself (Table 2) or to promote anti-MM immunity, whereby MoAbs target MM cell and immune cell interactions by acting as agonists or antagonists to key signaling receptors on NK and T cells (Table 3). Novel putative target antigens in MM are reviewed elsewhere [95].

#### 5.2.1. MoAbs Targeting the MM Plasma Cell

Arguably one of the most exciting new drugs on the MM clinical scene is daratumumab, a human anti-CD38 IgG1k mAb. Xenograft mouse models were used to compliment* in vitro* data that daratumumab induced apoptosis of MM cells [96], and the drug has subsequently progressed from phase 1/2 trials [97] to promising results in phase 3 trials [98, 99]. Returning to xenograft models has further helped to establish mechanisms of action—in addition to antibody-dependent cellular cytotoxicity (ADCC), daratumumab induces programmed cell death via Fc*γ* receptor-mediated cross linking [100]. They have also been useful to provide evidence for the effectiveness of combination therapy with lenalidomide prior to phase 3 trials (in previously lenalidomide/bortezomib resistant MM) [101] and ATRA via upregulation of CD38 expression [102, 103].

Also utilizing plasma cell CD38 and CD138 expression, alpha-radioimmunotherapy delivers localized radiation by delivering *α*-particles to target cells and has been developed to treat low level residual disease in MM. Effectiveness with minimal toxicity has been shown in the 5T mouse model with an anti-CD138 mouse antibody [104–107] and an anti-CD38 mouse antibody [108, 109] coupled to bismuth-213. A small dosimetry study in humans has shown feasibility of this therapeutic approach with good biodistribution in the BM [110].

Elotuzumab is an agonist for the signaling lymphocytic activation molecule-F7 (SLAM-F7, a.k.a. CS1). It enhances NK cell-mediated ADCC of CS1-expressing myeloma cells via IL-2 and TNF*α* pathways [111]. Elotuzumab proceeded to phase 1 clinical trials after* in vitro* and* in vivo* studies indicated enhanced NK cell antimyeloma activity, which was further augmented in combination with bortezomib [112]. Whilst tolerated well by RRMM patients, this mAb was ineffective as monotherapy [113], but clinical responses were seen when combined with IMiDs [114–116] or bortezomib [117, 118]. It is likely that the timing of administration and choice of combination therapy are important, as coadministration of dexamethasone is profoundly immunosuppressive to NK cells [7]. Researchers are now returning to mouse models to support phase III trial combination therapies and to further evaluate mechanism of action.

#### 5.2.2. Agonistic MoAbs

The cytotoxic functions of NK cells are regulated by a balance of expression of activating and inhibitory receptors, with the latter being known as killer cell immunoglobulin-like receptors (KIRs). The expression of ligands to KIRs is upregulated on MM cells, causing inhibition of NK cell activity [119]. IPH2101 is an anti-KIR human IgG4 mAb that prevents inhibitory KIR-ligand interaction against KIR2DL-1, KIR2DL-2, and KIR2DL-3. Initial* in vitro* experiments using IPH2101 in combination with lenalidomide showed synergistic anti-MM activity by enhancing NK cell function, and an* in vivo* tumor cell rejection model in C57BL/6J mice showed that a murine anti-KIR and lenalidomide had an additive effect [120]. Phase 1/2 clinical trials followed in humans with RRMM as monotherapy [121] and in combination with lenalidomide [122]. IPH2101 is no longer in development and has been superseded by another anti-KIR mAb lirilumab, which is in phase 1 trials in solid tumors.

Urelumab is an agonist for CD137, a costimulatory receptor target that is expressed on activated T cells, NK, and NKT cells. Activation with an agonistic mAb (4-1BB) exerted variable antimyeloma activity in Vk*∗*MYC mice [61, 109] and 5TGM1 mice [110]. In 5TGM1 mice, anti-CD137 mAb treatment led to a significant reduction in monoclonal paraprotein and extramedullary disease after 30 days of treatment, but had little effect on skeletal involvement [123]. It has also been trialed by two separate groups with two different transplant clones of Vk*∗*MYC: anti-CD137 mAb treatment with the Vk*∗*MYC 12653 clone showed a marked response in plasma cell infiltrate and paraprotein accompanied by a significant increase in survival [23], whereas the Vk*∗*4929 clone was virtually unaffected, even in combination with anti-CD40 antibody [124]. Of note, combination therapy with anti-CD137 and anti-CD40 prolonged survival in a minor proportion of treated mice who had a lower burden of disease at commencement of treatment: this highlights a problem with using transplant models with highly proliferative disease (as opposed to the indolent transgenic models), in that there may not be an opportunity for immunotherapies to be able to be shown to exert an effect. A phase 2 trial in RRMM patients with urelumab in combination with elotuzumab is underway (NCT02252263).

In order to promote immune synapse formation between T cells and tumor cells, bispecific T cell engager (BiTE) antibodies have been developed, which have had clinical success in lymphoma and acute lymphoblastic leukaemia. In myeloma, a xenograft model was used to provide* in vivo* data showing the efficacy of a CD3-BCMA BiTE [125], which is now in phase 1 studies in humans (NCT02514239). Other BiTEs in development include CD3-FcRH5, which has also progressed to phase 1 trial (NCT03275103), and an NK receptor binding BiTE CS1-NKG2D [126].

#### 5.2.3. Antagonistic MoAbs

A MM cell line J558L was used in one of the first* in vivo* experiments with BALB/c mice to demonstrate the antitumor efficacy of PD-L1 blockade [127]. In the 5T33 mouse model, as has been reported in human MM patients [128–131], PD-L1 is overexpressed on MM cells and PD-1 expression is increased on T cells [132, 133]. After the success of PD1/PD-L1 pathway blockade in melanoma, these inhibitors were used in an array of cancers but with underwhelming responses in phase 1/2 trials in RRMM [134, 135], and there has been some critique about the appropriateness of PD-1 inhibition in MM patients [41]. Chronically exhausted T cells may not have the capacity to respond to checkpoint blockade owing to a stably differentiated epigenetic landscape [136–138]. Alternately, it has since been demonstrated in human MM that hyporesponsive CD8^+^ T cell clones exhibit low expression of PD-1 or CTLA-4, suggesting that these cells are senescent rather than exhausted [139].

Returning to mouse models, inhibition of PD-1 had no effect on disease progression in Vk*∗*MYC [23]; however, in the 5T33 model, PD-1 was increased on T cells after autologous BM transplant and PD-L1 blockade increased efficacy of DC vaccine in combination with ASCT [132]. Further, PD-L1 mAb administered during the homeostatic proliferation phase after nonmyeloablative total body irradiation resulted in increased survival [140]. Immune checkpoint blockade with PD-1 blocking antibodies in the posttransplant setting also significantly improved disease control in Vk*∗*MYC mice [47].

To understand why PD-1 inhibition might be efficacious in these circumstances, it is important to note that PD-1 is not only upregulated in exhausted T cells but also as a normal process in effector T cells after activation of the T cell receptor. A balance between stimulatory and inhibitory signaling ultimately controls the magnitude of a T cell proliferation to antigen, and PD-1 facilitates apoptosis in CD8^+^ T cells by increasing reactive oxygen species [141]. Therefore, utilizing PD-1 inhibition in the post-ASCT setting could represent a unique timepoint at which derepression of proliferating T cells could enable superior clearance of tumor by myeloma-specific T cell clones.

However, recent studies have suggested a more complex role of PD-1 in T cells. PD-1 signaling causes a metabolic switch from glycolysis to lipolysis and fatty acid oxidation that is critical for the development and maintenance of T cell memory [142, 143]. This might suggest that PD-1 inhibition at T cell activation might impair the subsequent development of T memory cells, but this has not been reported with mouse models of acute viral infection [144–146], and further investigations in the MM setting are warranted.

Ipilimumab targets cytotoxic T-Lymphocyte antigen 4 (CTLA-4), another inhibitory receptor that is upregulated early in T cell activation. Human trials with ipilimumab have been in solid cancers, largely advanced melanoma, with some success but there are concerning, and potentially severe, immune-related adverse effects. This reiterates a problem with checkpoint blockade in that reverting evolutionarily acquired mechanisms that prevent the expansion of autoimmune T cell clones can result in autoimmune complications.

T cell immunoglobulin and ITIM domains (TIGIT) have recently been described as another effective immune checkpoint target in the Vk*∗*MYC mouse model [47, 147].

#### 5.2.4. Combination Therapy

It would seem a logical rationale to combine stimulatory and inhibitory checkpoint blockade, or NK and T cell checkpoint blockade, to maximally antagonize tumor-induced immune suppression. Indeed, there are a number of ongoing human trials with PD-1/PD-L1 inhibitors in combination with other immunotherapies, and with checkpoint blockade combinations that combat both NK and T cell inhibition (Table 3). Unfortunately, phase 3 trials using pembrolizumab in combination with IMiDs and dexamethasone have been suspended because of fatalities related to immune-mediated pneumonitis in the pembrolizumab-receiving groups. This could dampen the pharmaceutical appetite for further trials in MM with this combination.

Further studies with mouse models have been supportive of combination therapies. In the Vk12653 (4-1BB-responsive) transplant model, CD137 agonist treatment both reduced the proportion of Tregs and increased CD8^+^ effector frequency and function but also upregulated PD-1 and TIM-3 expression. Consequently, combination of CD137 mAb and anti-PD-1 early after BM transplant proved superior in MM disease control [44]. In the 5T33 mouse model, tumor-bearing mice treated with low dose whole body irradiation and combinations of immune checkpoint blockade (PD-L1 blockade with LAG-3, TIM-3, or CTLA4 blocking antibodies) had not only significantly improved survival rates, but also correlated with increased frequency of tumor-reactive T cells and elevated levels of inflammatory cytokines [133].

Ongoing work with checkpoint inhibitors is likely to concentrate on the timing of administration around other anti-MM therapies (particularly in the lymphopenic after BM transplant setting) and their use in combination with DC vaccines or oncolytic vaccines to optimize a specific anti-MM immune response. The potential to combine oncolytic vaccination (reviewed recently [148]) with immunotherapies to enhance immune surveillance was shown in a breast carcinoma mouse model with anti-4-1BB [149]. Specific to MM, the efficacy and safety of a vaccinia virus were established in a mouse xenograft model of MM [150], but has not yet progressed to human trials.

### 5.3. Immunomodulatory Drugs (IMiDs)

Thalidomide, or its analogs lenalidomide and pomalidomide, is often used in combination therapy with proteasome inhibitors, alkylating agents, and/or corticosteroids in the treatment of human MM. IMiDs were first introduced as an antimyeloma therapy without fully comprehending their mechanism of action. It is now understood that the binding of IMiDs to cereblon (CRBN) [151] leads to the degradation of two zinc finger transcription factors: IKZF1 and IKZF3 [152, 153]. This inhibits MM growth as IKZF1 is required for plasma cell maturation and loss of IKZF1/3 leads to decreased* IRF4 *and* MYC* expression [151, 154]. IMiDs achieved their name by being stimulatory to NK and T cells* in vitro*. This seems to be, in part, due to enhanced T cell IL-2 production, explained by the inhibition of IFZK1/3-mediated repression of the* Il2* promotor [152], although this effect is significantly abrogated by high dose steroid therapy [7].

It has long been appreciated that thalidomide does not have the same antitumor or antiangiogenic effect in rodents as that seen in humans [155]. Rodents have a point mutation in the substrate recognition protein of CRBN meaning that IMiDs cannot bind [156, 157] and therefore do not exert a direct antimyeloma effect in murine MM [19, 158]. To this end, several groups have developed humanized CRBN mouse models to further elucidate the* in vivo* immunomodulatory effects of IMiDs. It is possible that IMiDs have targets other than CRBN: there are a number of murine studies showing that lenalidomide enhances CD4^+^ T cell [159] and NK cell [85] antitumor activity and, in CB17-SCID mice bearing subcutaneous MM.1S plasmacytomas, pomalidomide-resistant xenografts could respond to lenalidomide despite CRBN levels being low [160]. IMiDs have also been shown to exhibit synergistic effects in combination with tumor-antigen loaded DCs in the MOP-315 murine model of MM [85, 161].

### 5.4. Small Molecule Inhibitors

Small molecule inhibitors generally exert their antitumor effects by promoting tumor cell apoptosis or cell cycle arrest but, somewhat serendipitously in some cases, their off-target effects on the immune system are beginning to be comprehended. In fact, some would say that their full therapeutic effect may* depend* on a functioning immune system [162].

Histone deacetylase inhibitors (HDACi) exert their full effect in murine tumors when combined with traditional chemotherapy [162] or with CD137 and CD40 mAb (that promote APC function and thereby support cytotoxic T cells) [163]. The importance of host-derived IFN*γ* for the effectiveness of HDACi has been demonstrated utilizing immunocompromised and immunocompetent mouse models of adenocarcinoma, aggressive lymphoma [162], and breast carcinoma [164].

Combination therapies using HDACi with DNA methyltransferase inhibitors or IMiDs are increasingly being studied. Panobinostat in combination with azacitidine has shown efficacy in the transplant Vk*∗*MYC model, but the role of the immune system was not evaluated [165]. Quisinostat in combination with decitabine in 5T33MM diseased mice was also favorable and is, at least partly, attributable to a significant induction of a type I interferon response; decitabine in particular resulted in increased DC maturation [166]. In a leukaemia mouse model decitabine was also reported to deplete MDSCs [167], whether that bears out in the MM tumor microenvironment is yet to be proven.

We await the long-term outcomes of phase 2/3 trials using Vorinostat and Panobinostat in RRMM in combination with bortezomib and/or IMiDs. Of note, some HDACi have been reported to reduce cereblon and so might be expected to impair the efficacy of IMiDs in this setting [168].

The inhibitor of apoptosis (IAP) antagonist LCL161 competitively inhibits binding of cellular IAPs, which are frequently inactivated in MM. Contrary to expectations, LCL161 reduced tumor burden in Vk*∗*MYC aged transgenic mice and transplant models. This was, again, shown to be the result of type I interferon production by the MM cells that resulted in their increased phagocytosis by macrophages [169]. A phase 2 clinical trial in humans did not show any response to single agent LCL161. Returning to the transplant Vk*∗*MYC model, the combination of LCL161 and anti-PD-1 was curative in all mice that completed 2 weeks of treatment. Hence combination therapy with LCL161 and PD-1 inhibition has been taken forward to phase 2 clinical trials (NCT03111992).

In a somewhat divergent approach to proinflammatory immunotherapies, bromodomain inhibitors (BETi), which are considered immunosuppressants due to their ability to reduce key proinflammatory cytokine and chemokine genes in sepsis [170], have been utilized in MM. The bromodomain inhibitor JQ1 resulted in rapid paraprotein regressions and improved survival outcome in transplanted Vk*∗*MYC mice, and it was shown to diminish IFN*γ*-induced PD-L1 expression on human and mouse tumor cell lines [171]. This is particularly relevant to myc-driven malignancies, as induction of PD-L1 may be partly due to the direct binding of MYC to the promoter region of CD274 (PD-L1) [172]. However, the JQ1 response was shown to be caused by the displacement of a BET protein from the transcriptional start site of CD274 and is therefore myc-independent [171]. Nevertheless, BETi may prove to have a role in selected human MM cells that have upregulated PD-L1 expression.

## 6. Future Directions

### 6.1. Personalized Care: Humanized Mice and 3D Culture Systems

Medical oncology is increasingly headed towards personalized care and, rather than a “one drug fits all” approach, it would be ideal to test the efficaciousness of immunotherapeutic drugs in an* ex vivo* model of an individual's tumor microenvironment prior to administration to the patient. Humanized mice may offer a conduit for this purpose, although it is not possible to incorporate a human thymus for normal T cell development: this may not be a disadvantage in the setting of MM due to the occurrence of thymic atrophy in immunosenescence.

Also promising are 3D tissue culture systems, which have the potential to be cheaper, less time consuming, and more ethically viable and have higher drug throughput than mouse models. The notable disadvantages currently (compared with mouse models) include the lack of vasculature and the challenges of maintaining plasma cells in an* in vivo*-like microenvironment alongside normal BM cell maturation. Several groups are making progress with replicating the BM microenvironment [173–179]: these generally involve either a tissue scaffold of osteoblasts [173, 174], crosslinked fibrinogen [176], or differentiated mesenchymal stromal cells [177] that can be combined with microfluidic chamber so that drug can be circulated similarly to capillary flow in the bone marrow. Increasing investment in these technologies over the last decade are likely to see improvements in the extracellular matrix scaffold and oxygen and nutrient distribution, as well as increased throughput and standardization of microscopic analysis and cell measurements.

### 6.2. Targeting Immunotherapies to Immune Profile

For some time it has been appreciated that evolving and cumulative genetic changes contribute to increased resistance of MM cells to apoptosis, the development of drug resistance, and poorer prognosis [71]. In some patients, clonal tides of MM can mean that therapies need to be switched depending on the dominant clone and its responsiveness [180]. In the same way, we should look to fitting treatments not only to the cytogenetic profile of the patients, but also to their immune profile.

Immune profiling can be performed by the assessment of T cell phenotype by flow cytometry; in one study, a putative immune signature by flow cytometry was associated with PFS and OS for MM patients treated with ASCT [181]. Features such as a reduced CD4:8, low proportions of circulating T_N_ and high proportions of T_EM_/T_EMRA_ indicate immunosenescence and shifts in the T cell population due to iatrogenic lymphopenia, and are likely to correlate with poorer responses to immunotherapeutics. Individuals with an immune profile comparable to healthy donors (i.e., younger, newly diagnosed MM with less advanced or smoldering phenotype of disease) are likely to achieve the greatest benefit from immunotherapeutics, and targeting this group in clinical trials may result in superior trial outcomes and greater cost-effectiveness.

### 6.3. Immunotherapy in Immunosenescence

If the adaptive immune system is essentially considered terminally differentiated or “burnt out” in immunosenescent, heavily treated MM patients, is there a role for immunotherapies at all? In such patients, perhaps alternative approaches to replenishing an effective T cell pool should be evaluated such as “off the shelf” CAR-T cells (derived from young healthy donors). Notably, in the 5T33 mouse model, it was shown that T lymphocytes from younger mice were associated with better disease control [182]. Thymic regeneration techniques [183, 184], whilst still some way from being utilized clinically, represent another solution to the diminished naïve T cell population.

Other immunomodulatory approaches also need to be considered such as mimicking or enhancing CD4^+^ T cell help [185]. The former might include cytokine support and agonists of costimulatory pathways such as CD27, and the latter utilizes innate immune signals to aid DC priming of CD8^+^ T cells. Of note, NK-like T cells are more frequent at extremes of age and are correlated with healthy ageing [186, 187]—further understanding of their potential plasticity will help with the development of age-appropriate immunotherapies.

## 7. Conclusions

Mouse models will continue to be important for selecting drugs for clinical trials, as the actual efficacy and toxicity cannot be predicted* in vitro*. However, moving away from utilizing hardy human MM cell lines in immunocompromised mice and, instead, trialing immunotherapeutics in the immunocompetent mouse are likely to yield more informative preclinical information for both the use of immunotherapeutics and enhancing the performance of small molecule inhibitors. Importantly (and with particular relevance to combination immunotherapies), acknowledging the complimentary roles of the innate and adaptive immune systems, and dendritic cells as the interface between the two, will be integral in furthering the success of immunotherapies.

## Figures and Tables

**Table 1:** Mouse models of multiple myeloma.

Model	Features	
**Xenograft models**		

SCID	Lack T and B lymphocytes	

NOD/SCID	SCID + no circulating complement and low NK cell function	

NSG	NOD/SCID + lack IL-2	
(NOD/SCID/IL2R*γ*^null^)		

SCID-hu	SCID implanted with human fetal bone chips	

SCID-rab	SCID implanted with rabbit bone chips	

SCID-synth-hu	SCID implanted with 3D polymeric scaffolds coated with human BM stromal cells	

**Immunocompetent models**		

5T series	Syngeneic transplant of cell lines from spontaneously arising MM in aged C57BL/KaLwRij mice[188, 189]	
	5T2MM	Moderate, progressive disease course
	5T33MM	Aggressive, rapidly progressive disease course
	5TGM1	Cell line derived from 5T33MM

Vk^*^MYC	*Transgenic: *spontaneous AID-dependent activation of MYC in post germinal B cells[17]	
	*Transplant*: syngeneic transplant of plasma cell lines from transgenic Vk^*^MYC mice	

Myc/Bcl-X_L_	Bitransgenic offspring of hemizygous Myc transgenic mice and hemizygous Bcl-X_L_ mice[17]	

XBP-1	*Eμ-*directed expression of XBP-1 spliced isoform, a factor governing plasma cell development that has been reported to frequently be overexpressed in human MM[18]	

MOPC315.BM	Syngeneic transplant of plasmacytoma-resembling MM cells from granulomas in Balb/c mice injected intraperitoneally with mineral oil	

**Table 2:** Translational studies with immunotherapeutics targeting myeloma cells. Dara: daratumumab, len: lenalidomide, dex: dexamethasone, bort: bortezomib.

Target	Pre-clinical evidence	Phase 1/2 trials	Phase 3 trials
B2M	Anti-B2M Ab (xenograft) [190]	*Not progressed to human trials*	

BCMA	CD3-BCMA BiTE (xenograft) [125]	NCT02514239	
	CAR-T (xenograft) [78, 79]	11D5-3-CD828Z[80]	
	bb2121(contains 4-1BB) [81]	NCT02658929	

CD38	Daratumumab causes MM cell apoptosis in xenograft models [191]	Phase 1: GEN501[96]	Dara/len/dex[98]
		Phase 1/2: SIRIUS[97]	Dara/bort/dex[99]
	*α*-radioimmunotherapy (5T33)[108]	*Not progressed to human trials*	

CD138	*α*-radioimmunotherapy (5T33)[104–107]	Phase 1 dosimetry study[110]	
	CAR-NK cells (NOD-SCID xenograft)[82]	*Not progressed to human trials*	

CS1	CAR-NK cells (NSG xenograft)[83]	*Not progressed to human trials*	

FcRH5	CD3-FcRH5 BiTE (xenograft)[192]	NCT03275103	

VLA-4	(5TGM1)[193]	*Not progressed to human trials*	

**Table 3:** Translational studies with immunotherapeutics targeting T and NK cells in the tumour microenvironment. Elo: elotuzumab; bort: bortezomib; dex: dexamethasone; thal: thalidomide; pom: pomalidomide; dara: daratumumab; CTD: cyclophosphamide, thalidomide, dexamethasone; NDMM: newly diagnosed multiple myeloma; RRMM: relapsed/refractory multiple myeloma.

Target	Pre-clinical evidence	Phase 1/2 trials	Phase 3 trials
CD137 (4-1BB)	Vk^*^MYC[23, 124]	NCT02252263: Urelumab (+ elotuzumab)	
	5TGM1[123]		

CS1 (SLAMF7)	Anti-CS1, bortezomib (xenograft) [112]	Phase 1: Elo monotherapy[113]	NCT01335399 (ELOQUENT-1): Len/dex +/- elo in NDMM
		Phase 1: Elo/bort[117]	
		Phase 2: Elo/bort/dex[118]	NCT01239797 (ELOQUENT-2): Len/dex +/- elo in RRMM[194]
		Phase 1: Elo/len/dex[114]	
		Phase 1b/2: Elo/len/dex[115]	ACTRN12616001030460 (MM20): Elo/CTD vs CTD in RRMM
		Phase 2: Elo/thal/dex[116]	

CTLA-4	CTLA-4 Ig (Vk^*^MYC)[23, 195]	NCT01592370 Arm 2: Ipilimumab (+ Nivolumab)	

KIR ligands	Anti-murine KIR mAb + len (xenograft)[120]	Phase 1: IPH2101 monotherapy[121]	
		Phase 1: IPH2101 + len[122]	
	
		NCT02252263: Lirilumab (+ Elotuzumab)	
		NCT01592370 Arm 2: Lirilumab (+ Nivolumab)	

PD-1	PD-1 (Vk^*^MYC)[23]	Phase 1b: Nivolumab[134]	NCT02576977 (KEYNOTE 183): Pom/dex +/-
		Pembrolizumab/len/dex in RRMM[196]	pembrolizumab in RRMM
		Phase 1/2: Pembrolizumab/pom/dex[135]	
			NCT02579863 (KEYNOTE 185): Len/dex +/-
		NCT01592370 Arm 3/4: Nivolumab + dara +/- pom/dex	pembrolizumab in NDMM
		NCT02036502 (KEYNOTE-023): Phase 1	

PD-L1	PD-L1 (5T33)[132, 140]	NCT02685826: Durvalumab/len +/-dex	
		NCT02616640: Durvalumab/pom +/-dex	
		NCT02807454: Durvalumab +/-dara	
		NCT02431208: Atezolizumab +/-dara	
